# Functional and Clinical: An Explainable Deep Learning Model for Multimodal Alzheimer's Disease Classification

**DOI:** 10.1002/brb3.71240

**Published:** 2026-01-30

**Authors:** Samuel L. Warren, Ahmed A. Moustafa

**Affiliations:** ^1^ School of Psychology, Faculty of Society and Design Bond University Gold Coast Queensland Australia; ^2^ Department of Human Anatomy and Physiology, Faculty of Health Sciences University of Johannesburg Johannesburg South Africa

**Keywords:** Alzheimer's disease (AD), clinical data, deep learning, default mode network (DMN), explainable artificial intelligence (XAI), functional magnetic resonance imaging (fMRI)

## Abstract

**Purpose:**

Functional magnetic resonance imaging (fMRI) and deep learning models can classify Alzheimer's disease (AD) with high accuracy. These models are highly adaptable and work with a plethora of architectures, data types, and AD stages. However, fMRI deep learning models lack clinical application due to issues with small datasets, explainability, and reliability (e.g., data leakage).

**Methods:**

In this study, we address these issues using multimodal and explainable artificial intelligence (XAI) methods. Specifically, we overcome data size limitations by supplementing fMRI data with clinical tests, use a strict leave‐one‐out cross‐validation regime to control for data leakage, and apply perturbation ranking to explain the importance of features in our model. Our 3D convolutional neural network model was trained and validated on 52 participants from ADNI using five clinical tests and fMRI of the default mode network.

**Findings:**

The resulting multimodal model classified AD from controls with an accuracy of 90% and outperformed the same architecture without clinical data (58% accuracy). Our feature rankings showed that clinical tests changed in importance within our model depending on the diagnostic group. For example, our model found the MoCA to be highly important for classifying controls but not for AD. This trend of feature importance was seen across almost all fMRI and clinical features.

**Conclusion:**

Our model was highly accurate and highlighted the importance of combining fMRI and clinical data for AD classification. These findings have implications for the refinement of multimodal deep learning models; however, our small sample and need for external validation are also noted.

## Graphical abstract

A multimodal Alzheimer's classification pipeline that combines clinical tests with fMRI networks to output individual‐level predictions and variable importance metrics.

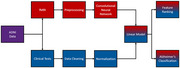

## 1 Introduction

The development of accurate and timely diagnostic methods is required to combat Alzheimer's disease (AD; Rasmussen and Langerman 2019). A large portion of the AD literature is responding to this need by pairing AD biomarkers with cutting‐edge classification models (Arya et al. 2023; Bhandarkar et al. 2024). For example, many studies have achieved high diagnostic accuracy using neuroimaging biomarkers and artificial intelligence classification models (Illakiya and Karthik 2023; Toumaj et al. 2024). One of these innovative methods uses functional magnetic resonance imaging (fMRI) and deep learning to detect AD (Warren and Moustafa 2023). These models generally work by learning functional changes in the AD brain to discern case samples from control samples. While fMRI and deep learning models are not the only viable methods, they do have some unique benefits. For example, functional biomarkers are sensitive to early‐stage AD, deep learning models are semiautonomous (Amiri et al. 2024), and fMRI is noninvasive (Fathi et al. 2022). Multiple studies have used deep learning and fMRI models to classify AD (Tajammal et al. 2023; R. Wang et al. 2023). These models are highly accurate and can classify multiple stages of the AD continuum (Alorf and Khan 2022; J. Zhang et al. 2024). Reviews have also highlighted that fMRI and deep learning are highly adaptable and can work with many different architectures, features, data formats, and study designs (Ebrahimighahnavieh et al. 2020; Rashid et al. 2020). Accordingly, fMRI and deep learning models could be a key method for improving AD diagnoses.

One primary issue with fMRI deep learning models is the lack of data for model training and validation (Fathi et al. 2022). This dearth of data is problematic as deep learning models will often have poor accuracy, reliability, and generalizability when using a restricted dataset (Keshari et al. 2020; Litjens et al. 2017). There are some strategies for addressing issues with data availability. For example, data augmentation, data generation, and transfer learning are common approaches (Brigato and Iocchi 2021; Santos and Papa 2022; Warren and Moustafa 2025). These methods generally work by supplementing a limited dataset with synthetic or similar data (Smucny et al. 2022). Multimodal deep learning models work on the same premise; however, rather than using synthetic or nondomain data, they bolster models with participant data from another modality (e.g., clinical tests). In the literature, some of the most accurate fMRI deep learning models incorporate data from other modalities (Ebrahimighahnavieh et al. 2020; Warren and Moustafa 2023). For example, a series of studies have combined fMRI with diffusion tensor imaging and structural MRI (sMRI; Meng et al. 2022; Song et al. 2021; Y. Wang et al. 2018; L. Zhang et al. 2021). These multimodal models are highly accurate, work on relatively small AD samples, and could help overcome problems with data accessibility.

Most multimodal fMRI models supplement their data with other neuroimaging modalities (Goenka and Tiwari 2021). However, deep learning is adaptable to many other types of data. One important area is the combination of fMRI deep learning models with clinical data. These models are less common in the literature but could have significant benefits for clinical application. For example, clinical data are widely available, are easy to collect, do not require significant analysis, and can pair well with machine learning architectures (e.g., support vector machines; Z. Li et al. 2021). Only a few studies have combined clinical data, fMRI, and deep learning for AD diagnosis. A study by Guo and Zhang (2020) paired participants’ clinical information—such as their APOE4 genotype, age, and sex—with fMRI time‐series data and an autoencoder model. Their resulting pipeline had an AD classification accuracy of approximately 90%–95%. Another study by Ghafoori and Shalbaf (2022) expanded on this multimodal concept and incorporated 16 different clinical measures into an fMRI deep learning model. Their model had an accuracy of 91.72% when predicting MCI to AD conversion and outperformed the same model without clinical data (87.67%). These studies highlight the potential of combining fMRI, deep learning, and clinical data; however, significantly more research is required to understand the reliability and feasibility of combining fMRI and clinical features. This study seeks to investigate these gaps in understanding to improve AD classification models.

It is important to note that multimodal models have some specific issues that could impede adoption (Fathi et al. 2022). First, combining multiple data sources per participant can result in data leakage (Yagis et al. 2021). We define data leakage as a scenario where classification results are biased due to the model accessing true diagnostic information during inference that would not usually be available (Kapoor and Narayanan 2023). For example, a study could encounter data leakage when a participant has multiple fMRI images that appear in both the model training and testing datasets. Such a scenario would mean that the model performs better than expected on the testing dataset because it is already familiar with that participant's data from training. Another, more field‐specific scenario could involve giving the model clinical data that were also used to determine participants’ diagnoses. For example, the Alzheimer's Disease Neuroimaging Initiative (ADNI) uses the Mini–Mental State Examination (MMSE) as part of its diagnostic methodology (Alzheimer's Disease Neuroimaging Initiative 2016). Thus, using the MMSE as a clinical measure in a deep learning model could bias the model through label leakage. In this article, we take a proactive approach to preventing data leakage by excluding ADNI's diagnostic tests from our dataset, concatenating (i.e., packaging) participants’ data, and adopting a strict form of cross‐validation (i.e., leave‐one‐out cross‐validation [LOOCV]).

Another issue with multimodal models is that they lack explainability (Holzinger et al. 2017). Most deep learning models are called black boxes because they do not provide information on their inner workings. This hidden characteristic of deep learning models can be permissible in some low‐risk circumstances. However, in the case of disease diagnoses, it is imperative that models can be understood and trusted (Martin et al. 2023). Such transparency can help combat bias by identifying irregularities in the dataset (e.g., data leakage), identifying key variables for AD classification, and improving confidence in models for clinical use (Viswan et al. 2024). We address some of these issues with model transparency using explainable artificial intelligence (XAI; Toumaj et al. 2025; H. Wang et al. 2025). Specifically, we use a feature ranking technique that calculates the importance of each clinical variable in our model. This approach enables us to understand how our clinical and functional features are used to classify participants. Moreover, feature ranking lets us investigate how multimodal models operate and whether this differs from more traditional diagnostic models (e.g., statistical models). Such a technique is important for creating accurate multimodal models and understanding the role of functional and clinical features in AD.

**FIGURE 1:**
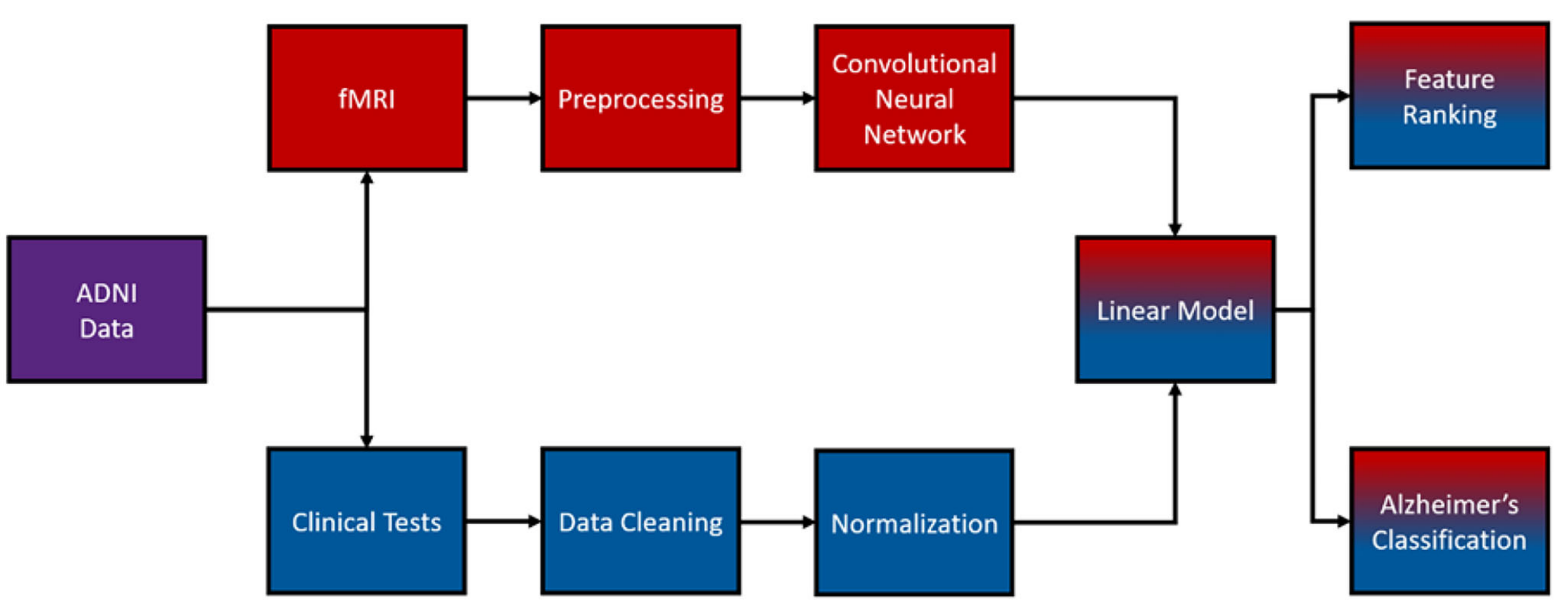
An outline of our multimodal pipeline. The color red denotes imaging data, blue indicates clinical data, and a mixture represents multimodal data.

In this study, we create a multimodal deep‐learning model that combines clinical data with fMRI to classify AD. This approach aims to harness the benefits of multimodal fMRI deep learning models while also addressing some of their common issues. Specifically, we seek to accurately classify a small AD sample while addressing issues with data availability, model explainability, and data leakage. We address these issues using methods such as LOOCV, data concatenation, and feature importance ranking. Feature importance ranking is especially important as this XAI technique helps extract information about the ability of each clinical feature in our model. As a result, this feature ranking should increase the explainability of our model and provide information on future directions for adapting and optimizing our methodology. We also create a unimodal model that only uses fMRI data for comparison. Along with feature importance ranking, this comparison model helps understand the impact of the clinical data on our fMRI deep learning model. Prior works have investigated similar topics, such as MCI to AD prediction and sMRI multimodal models (Ghafoori and Shalbaf 2022; Guo and Zhang 2020; Qiu et al. 2022). However, to our knowledge, this is the first model to classify AD from controls by combining fMRI, clinical tests, and explainable deep learning methods. This novel pipeline (see Figure 1) is important as it addresses current issues with small‐scale diagnostic models that are important for preparing them for real‐world applications. Based on the evidence above, we hypothesize the following:

Hypothesis 1 (H1) Our multimodal deep‐learning model will have a higher accuracy than our unimodal deep learning fMRI model.

Hypothesis 2 (H2) Clinical features will be among the most important features for AD classification (i.e., appear in the top 50% of feature rankings).

## 2 Methods

### 2.1 Participants and Data

Our data were acquired from the second ADNI cohort (ADNI2; Beckett et al. 2015). ADNI2 contains a wide range of neuroimaging, biological, demographic, and neuropsychiatric data for each participant. We extracted clinical tests, MRI (fMRI and sMRI), and demographic data for our analysis (Figure 1). Our sample was randomly selected from the wider ADNI2 cohort using a random number generator. This randomized strategy was chosen to create a balanced sample for deep learning. More participants are available in ADNI2; however, we could not increase our sample size due to missing data, resource limits, and the need for balancing. Our resulting sample contained 52 baseline participants (see Table 1) who were balanced across the AD (*n* = 26) and cognitively normal (CN) diagnosis groups (*n* = 26). Each of these participants contained four cross‐sectional fMRI images of default mode network (DMN) regions of interest (ROIs) (defined by seed‐based correlation) and six clinical measures, resulting in a total of 208 images and 520 datapoints for the study. The diagnosis for these groups was determined by ADNI physicians using the following assessments: A clinical interview, the Logical Memory II subscale from the Wechsler Memory Scale (Revised), the MMSE, the Clinical Dementia Rating scale, the National Institute of Neurological and Communicative Disorders and Stroke and the Alzheimer's Disease and Related Disorders Association Alzheimer's Criteria (NINCDS–ADRDA), and a general cognitive and functional assessment (Alzheimer's Disease Neuroimaging Initiative 2016; Beckett et al. 2015). These diagnoses were used as ground truth labels in our deep learning model. The diagnostic measures (e.g., MMSE) were also excluded from our clinical data to avoid label leakage. See Table 1 for our sample's descriptive statistics.

**TABLE 1:** Sample descriptive statistics.

Diagnosis	Group count	Age (mean)	Age (SD)	Sex (male/female)
AD	26	72.7	6.7	12/14
CN	26	77.1	7.6	10/16

Abbreviations: AD, Alzheimer's disease; CN, cognitively normal; SD, standard deviation.

### 2.2 Clinical Measures

Our clinical data contained six measures from five clinical tests. These measures were the Alzheimer's Disease Assessment Scale—Cognitive Subscale 13 (ADAS13; Mohs et al. 1997), the Everyday Cognition scale (ECog; Farias et al. 2011), the Functional Activities Questionnaire (FAQ; Pfeffer et al. 1982), the Montreal Cognitive Assessment (MoCA; Nasreddine et al. 2005), and Rey's Auditory Verbal Learning Test (RAVLT; Rey 1958). We used total scores for all neuropsychological tests, except for the RAVLT, where we used the immediate recall (0–15 words immediately recalled) and RAVLT percentage forgetting measures. We chose our clinical measures based on a mixture of methodological reasons and past evidence. First, most of these measures were used by Ghafoori and Shalbaf (2022) and helped increase model accuracy. Ghafoori and Shalbaf (2022) also included additional clinical measures; however, we decided to limit our clinical data and remove the diagnostic tests used by ADNI to create participant labels (as discussed above). The choice to use fewer tests was also influenced by our previous findings that only a handful of clinical tests were required to detect AD (Warren et al. 2021, 2024). Using fewer clinical measures also reduces the chance of label leakage and complicating our analyses (e.g., worse explainability and multicollinearity). All six neuropsychological measures were normalized so that they were all on the same scale (Figure 1). This normalization was calculated using the following formula:

$$X_{\mathrm{Normalized}}=\frac{X-X_{\mathrm{Minimum}}}{X_{\mathrm{Maximum}}-X_{\mathrm{Minimum}}}.$$

### 2.3 fMRI Preparation

To prepare for preprocessing, our MRI data (structural and functional) were converted from DICOM to Neuroimaging Informatics Technology Initiative (NIfTI) formatting using dicm2nii in MATLAB (X. Li et al. 2016) and formatted into brain imaging data structure (BIDS) using MRIcroGL. Our data were then preprocessed using CONN (release 22.a; Whitfield‐Gabrieli and Nieto‐Castanon 2012) using standard parameters. After preprocessing, we used CONN to perform first‐level analyses (i.e., feature extraction) on the fMRI data. CONN uses a seed‐based connectivity analysis for first‐level analysis that creates correlation maps of multiple ROIs (Whitfield‐Gabrieli and Nieto‐Castanon 2012). Our first‐level analysis resulted in correlation maps for 32 ROIs per participant. These files were each part of various common brain networks—such as the sensory–motor, visual, and language networks. Our study only used the four ROIs corresponding to the DMN for each participant. Specifically, we extracted ROIs for the posterior cingulate, left lateral parietal, right lateral parietal, and medial prefrontal cortices. We chose to only study the DMN because it allows for the best balance between model explainability and feature selection. That is, including multiple ROIs in the deep learning black box would remove our ability to understand which brain network our functional features come from and complicate comparisons with clinical features. We also chose the DMN because it has strong links to AD and cognitive decline (Grieder et al. 2018; Mondragón et al. 2021; L. Zhang et al. 2020). Participants’ DNN ROIs were grouped together to allow for simultaneous input into the model to avoid data leakage. This data concatenation was achieved by converting each ROI from an image to a tensor and then concatenating all ROI tensors together (i.e., as a tensor of tensors). This concatenation was achieved using a custom data loader in PyTorch.

### 2.4 Deep Learning Models

**FIGURE 2:**
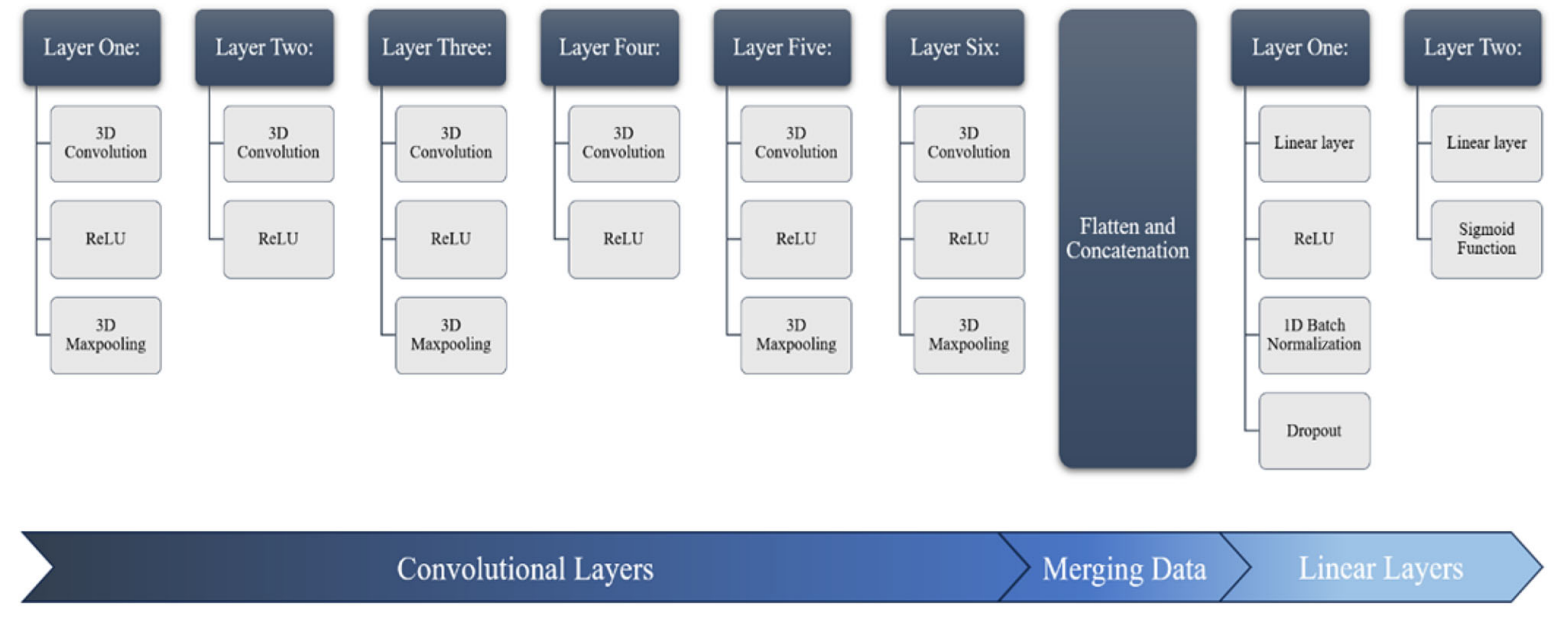
Summary of the model architecture. In the multimodal model, the convolutional and linear layers were separated into two models to facilitate feature ranking. The unimodal model contained all layers in one model.

This study involved the creation of two 3D‐CNN deep learning models. The first was our main multimodal model that used participants’ clinical and fMRI data to classify AD. We based our architecture on the work of Ghafoori and Shalbaf (2022), who conducted a similar study. However, our model contained some significant alterations (Figure 2). Notably, we split the architecture to facilitate feature ranking, reduced the number of neurons in each layer (to reduce overfitting), wrote a custom data loader, and rewrote the linear layers to include dropout and batch normalization. These parameters were altered due to differences in study design and data, as well as the difficulty of our classification task (i.e., overfitting). Our model took four fMRI images and six clinical test scores as input (these measures are outlined in Sections 2.2 and 2.3), resulting in 10 datapoints per participant and 520 total datapoints. Each participant's data were loaded together and simultaneously passed into the model. The fMRI data were passed to the model's convolutional layers, which learned and extracted relevant DMN features. The clinical data skipped the convolutional layers as they are only for processing higher‐dimensional data (e.g., images). Instead, the clinical data were concatenated with the DMN features that were extracted by the convolutional layers (see Figure 1). This concatenation occurred using PyTorch's concatenation function using the same methods we used to combine participants’ DMN images (see Section 2.3). The concatenated data were then passed to a linear model for further learning and classification. The model used the Adam optimizer and binary cross‐entropy (BCE) loss, and the linear model performed classification using a sigmoid activation function (Kingma and Ba 2017).

We used perturbation ranking to calculate the importance of features in our main multimodal model (Farahani et al. 2022). Perturbation ranking defines feature importance by systematically altering model inputs (e.g., randomizing integers) and calculating the impact of each variable's absence on classification performance. The ranking of each feature is defined after iterating through all possible feature perturbations and comparing the magnitude of their associated errors (i.e., impact on model performance). Importantly, our feature ranking function would only occur after the completion of a training and validation fold. That is, the feature ranks would be calculated using the model as it appeared in the final validation epoch. After the completion of all folds, the error of each perturbation iteration was averaged for each diagnostic group and ranked in order of importance (higher model error indicates greater feature importance). We used LOOCV for our training and validation regime to ensure strict data management and reliable results with a small dataset (Yates et al. 2023). We also used data augmentation (i.e., random image flipping and rotation) and dropout to reduce overfitting. All hyperparameters for our model can be seen in Table 2.

**TABLE 2:** Model hyperparameters.

Hyperparameter	Value
Epochs	80
Batch size	64
Random rotate probability	0.5
Random flip probability	0.5
Dropout	0.2
Resize transform	91, 91, 91
Learning rate	0.0001
Cross validation	LOOCV
Optimizer	Adam
Loss	BCE loss

*Note*: Hyperparameters were the same for the univariate and multivariate models.

After constructing our multimodal model, we created a second model that classified AD using just the fMRI data. This unimodal model was created to compare the difference in model performance when excluding clinical data. This comparison model used the same architecture and hyperparameters as the main multimodal model. The only difference was the exclusion of the clinical data and, thus, the reduction of input neurons in the first linear layer (see Table 3). For a fair comparison, we simultaneously tuned the hyperparameters of the unimodal and multimodal models on the first fold. That is, hyperparameters were only chosen if they increased the accuracy of both models. We chose this combined tuning strategy as focusing on one model could bias our comparison. Following tuning, we ran both models in their entirety (i.e., all LOOCV folds) and collected our results. We initially planned to perform a statistical analysis on the clinical data as another unimodal comparison. However, the sample size was not large enough to receive accurate results (as informed by a G*power sample size analysis for a logistic regression; Faul et al. 2007). The results of our multimodal and unimodal models are detailed in the following section.

**TABLE 3:** Layer input and output values.

Layers	Input	Output	Kernal	Stride
Conv1	4	8	6 × 6 × 6	1
Conv2	8	16	3 × 3 × 3	1
Conv3	16	24	3 × 3 × 3	1
Conv4	24	24	3 × 3 × 3	1
Conv5	24	32	3 × 3 × 3	1
Conv6	32	64	3 × 3 × 3	1
Lin1	70^a^	32	—	—
Lin2	32	1	—	—

*Note*: All max pooling layers had a stride of 2 and a kernel size of 3 × 3 × 3.

^a^ The linear inputs for the multimodal model increased from 64 to 70 due to the addition of the six clinical measures.

## 3 Results

### 3.1 Classification Results

**FIGURE 3:**
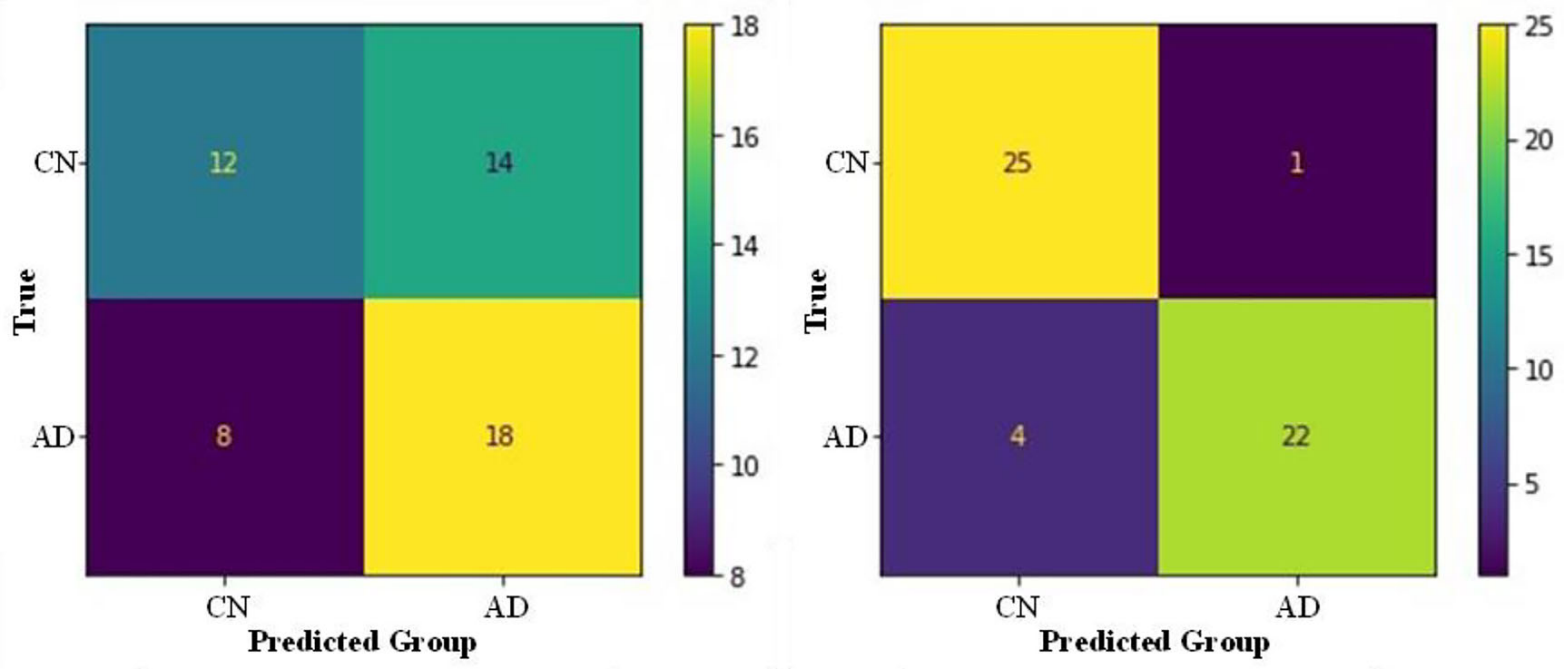
Confusion matrices for the unimodal and multimodal models. The colors in the confusion matrices indicate the frequency participants were classified correctly or incorrectly (split by true and predicted diagnoses).

Our multimodal model used fMRI of the DMN and clinical tests to classify AD from controls. The specific variables used were functional ROIs for the posterior cingulate, left lateral parietal, right lateral parietal, and medial prefrontal cortices. The clinical variables included ADAS‐cog 13, ECog, FAQ, RAVLT immediate, RAVLT percentage forgetting, and MoCA scores. These functional and clinical variables were analyzed using a 3D‐CNN. Our multimodal model had a classification accuracy of 90.38% (Figure 3). The model also had a sensitivity of 0.84, specificity of 0.96, precision of 0.96, and F1 score of 0.90. These metrics indicate that our multivariate model had a strong classification ability. This result was further confirmed by the Matthews Correlation Coefficient (MCC) of 0.81, which indicates a strong positive relationship between our model predictions and ground truth values (i.e., diagnosis labels). Accordingly, we could conclude that combining clinical tests and fMRI can produce an accurate diagnostic model.

Our unimodal model was created for comparison. This model has the same architecture and inputs, except for the exclusion of the clinical tests. This fMRI‐based unimodal model had an accuracy of 57.69% when classifying AD from controls (Figure 3). The model also had a sensitivity of 0.69, specificity of 0.46, precision of 0.56, F1 score of 0.62, and MCC of 0.16. These results indicate that our unimodal model has a weak classification ability that is marginally above chance. There is a 32.69% difference in classification accuracy between the multimodal and unimodal models. Accordingly, evidence suggests that the removal of clinical data results in a significant loss in accuracy. Therefore, we can accept our first hypothesis, which is that clinical data will increase the classification ability of an fMRI deep‐learning model in a constrained dataset.

### 3.2 Feature Ranking

**FIGURE 4:**
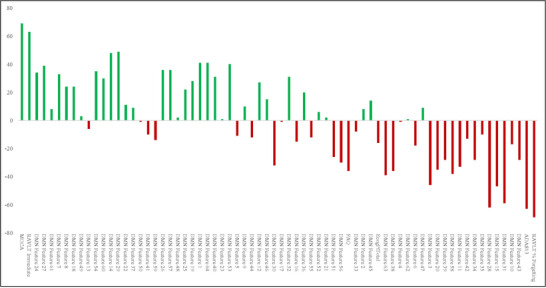
The change in feature rankings between groups. Change is calculated by subtracting a feature's AD rank from its CN rank. Drastic deviations in rank show that a feature is important for one diagnostic group but not the other. All features are in order of their original CN importance ranking. The colors highlight the direction of a rank change and the general splitting of features between the diagnosis groups.

We performed perturbation‐based feature importance ranking to understand our model. This perturbation method took all clinical and functional features as input and returned the model's error when each feature was randomized (see Section 2.4). The error values were averaged across LOOCV folds and used to calculate ranking and importance values. Importantly, we were also able to calculate feature rankings for each diagnosis group due to the nature of LOOCV (i.e., each fold's validation sample is one participant). The results of our feature ranking are shown in Table 4 and Figure 4. These results, shown in Table 4, highlight that features have different importance depending on the diagnostic group. For example, the MoCA was the most important feature when classifying CN participants but was the least important feature for classifying AD. The ranking of clinical features also followed this trend, where highly ranked features in one diagnosis group were at a low rank in the other (see Figure 4). Our second hypothesis predicted that we would see all clinical tests in the top 50% of all features. More interestingly, we observed that clinical tests were more nuanced and had differing importance depending on the diagnostic group. Thus, we rejected our second hypothesis. We discuss our results’ implications, strengths, and limitations in the following sections.

**TABLE 4:** Clinical test feature rank, importance, and error by diagnosis group.

Clinical test feature rank	CN group	CN error	CN importance	AD group	AD error	AD importance
1	MoCA	0.237	1.000	RAVLT % forgetting	0.209	1.000
2	RAVLT immediate	0.179	0.753	ADAS13	0.175	0.835
3	FAQ	0.141	0.595	FAQ	0.164	0.785
4	EcogPtTotal	0.140	0.590	EcogPtTotal	0.140	0.670
5	ADAS13	0.131	0.553	RAVLT immediate	0.116	0.556
6	RAVLT % forgetting	0.122	0.512	MoCA	0.091	0.434

*Note*: Feature importance was calculated using all features and is a value between 0 and 1, where 1 is the most important.

## 4 Discussion

In this study, we created a multimodal AD classification model using fMRI, deep learning, and clinical data. This model classified AD from controls with 90% accuracy, similar to other cutting‐edge multimodal models in the literature (Guo and Zhang 2020). Importantly, our multimodal model replicated the results of Ghafoori and Shalbaf (2022), showing the ability of multimodal fMRI and clinical test models to accurately classify AD and outperform our unimodal comparison model (32.69% lower accuracy without clinical tests). We suspected that the lower unimodal model accuracy occurred due to the lack of data in the model. For example, we only used four DMN images per person, while it is common for other fMRI studies to use more brain networks and features (e.g., Qureshi et al. 2019). Accordingly, the removal of clinical data could be detrimental, as just the DMN features may not be enough information to accurately classify such a small population. Similarly, we hypothesize that the clinical data can drastically improve the model because it can link to functional features from the DMN and give a more rounded view of AD pathology. For example, clinical data could help due to the known links between the DMN, autobiographical memory, and AD progression (Grieder et al. 2018; Mondragón et al. 2021; L. Zhang et al. 2020). Consequently, we suggest that clinical tests could be strong features that promote the extraction of strong DMN features.

Our multimodal model included XAI techniques to help understand how the model performed AD classification. Using perturbation, we were able to calculate the error, importance, and ranking of features in our model. We hypothesized that clinical features would be highly important (i.e., ranked within the top 50% of all features) for AD classification, thus explaining the disparity between unimodal and multimodal models. However, we observed that each clinical test had different levels of importance depending on the classification group. For example, the MoCA was the most important feature for classifying control participants but the least important for detecting AD. This finding is interesting as the MoCA is very sensitive to the early stages of AD (e.g., MCI) and, thus, could be a strong discriminator of difficult CN cases (Costa et al. 2014). This observed disparity between AD and CN features was seen across the whole feature set, with those important for classifying one group having less relevance for the other. These results indicate that our model is not just learning features that can discriminate AD from controls. Instead, our model appears to collect separate and complex feature maps for recognizing each diagnosis group. Such a finding highlights clinical tests that are potentially sensitive to one condition more than another, which is critical for early AD diagnosis. Our results also show the importance of XAI and the richness of information that can be extracted from transparent models. This information is important as it can be used to create more efficient and clinically applicable models (Farhoudian et al. 2025). For example, in a future iteration of our model, we could remove features with lacking importance (e.g., ECog) and test the importance of new AD measures (e.g., APOE4). In turn, XAI can help make highly accurate and efficient models through iteration and refinement.

This study had some notable strengths and limitations. One key limitation is that the generalizability of our multimodal feature ranking methodology is unknown. Individual methods like perturbation, LOOCV, fMRI, and deep learning are not new; however, the combination of all these techniques results in new approaches that lack clear guidelines. For example, there is no clear advice on how feature ranks should be calculated in an LOOCV design. Accordingly, it would be improper to suggest that our feature rankings generalize to all models and that our findings are directly meaningful for clinical diagnoses. It is highly important to stress that the conclusions from feature ranking should only be considered in the context of our model unless substantially replicated. For this reason, we have attempted to be as transparent and rigorous as possible. Nevertheless, the reliability and generalizability of our methodology are yet to be determined. Another limitation is the small sample size of 52 participants and 520 data points. We suggest that the replication and expansion of Ghafoori and Shalbaf (2022) and the usage of data augmentation bring validity to our method; however, there is still a potential lack of generalizability and sample bias that can come with such a small sample. In turn, our methods require further validation on larger and external dementia datasets and should not be used to inform clinical decision‐making.

A key strength of our study is our model's ability to classify a small AD sample using 10 multimodal variables (four DMN images and six clinical measures). Deep learning is an important technology for disease diagnosis; however, it often lacks the ability to perform well with small samples. Our multimodal model shows that pooling a participant's data can help to classify AD, even in a small sample. This finding is advantageous for the application of deep learning models to small, real‐world, and edge‐case scenarios like AD diagnoses in local communities (Amiri et al. 2023). Our usage of XAI is also a considerable strength. By harnessing feature ranking methods and incorporating them into a 3D‐CNN, we could understand how clinical data interact with functional features and help with AD classification. This design has allowed us to directly measure and visualize the impact of features in our model that would otherwise be unknown. In turn, our feature ranking results showed the importance of using explainable and multimodal techniques when classifying complex, multifaceted disorders like AD. Lastly, our steps to prevent data leakage are a notable strength. We use a custom fMRI‐compiling data loader to avoid leaking participants’ information across samples, employ LOOCV to ensure strict data management, and exclude the clinical measures associated with participants’ ground‐truth diagnoses. Such methods for combating label leakages are rarely used in the literature and should garner more focus.

Our results and methodology highlight many opportunities for future innovation. We suggest that future research seek to explore the plethora of AD biomarkers and measures that could be used in a multimodal deep learning model. For example, harnessing clinical biomarkers, like tau, could be important for making accurate and accessible AD diagnosis models. This future research should also seek to create models that use AD data that are widely available and accessible. We understand that neuroimaging methods are not universally available and that model accessibility is just as important as model accuracy. Accordingly, future research should seek to make multimodal AD classification models that can work in settings with restricted resources (e.g., low‐income countries, local clinics, and rural medicine). Building on this multimodal approach, we suggest that it is equally important that future models incorporate XAI techniques, like feature ranking. There are many XAI approaches that could be used, such as convolutional feature mapping. The specific techniques may require adjustment to work with neuroimaging models and multimodal data. Nevertheless, harnessing these explainable methods will be key to making trusted and generalizable models.

In this article, we present a novel approach to combining and interpreting multimodal AD data. This approach led to a highly accurate AD classification model that works on a small population. The resulting model also uncovered some potential relationships between clinical tests, functional imaging features, and AD diagnoses. While still an emerging technique requiring significant development, we believe that our results are a positive step in the direction of clinical screening and automated dementia detection models. Specifically, we believe that our explainable and multimodal approach is important for the future of dementia diagnoses, as the condition itself is largely unknown and multifaceted. We hope that future research builds on our findings and that we, as a community, can help to improve the detection of AD.

## Author Contributions

**Samuel L. Warren**: conceptualization, data curation, formal analysis, investigation, methodology, project administration, resources, software, validation, visualization, writing – original draft, writing – review and editing. **Ahmed A. Moustafa**: conceptualization, funding acquisition, supervision, validation, writing – review and editing.

## Funding

S.W. would like to acknowledge funding received as part of the Australian Government's Research Training Program Scholarship. Data used in the preparation of this article were obtained from the ADNI database (adni.loni.usc.edu). As such, the investigators within the ADNI contributed to the design and implementation of ADNI and/or provided data but did not participate in the analysis or writing of this report. A complete listing of ADNI investigators can be found at http://adni.loni.usc.edu/wp‐content/uploads/how_to_apply/ADNI_Acknowledgement_List.pdf.

## Ethics Statement

Ethical approval was received from Bond University prior to conducting this study.

## Conflicts of Interest

The authors declare no conflicts of interest.

## Data Availability

The data for this study were acquired from ADNI. ADNI data collection and sharing for this project were funded by ADNI (National Institutes of Health Grant U01 AG024904) and DOD ADNI (Department of Defence award number W81XWH‐12‐2‐0012). ADNI is funded by the National Institute on Aging, the National Institute of Biomedical Imaging and Bioengineering, and through generous contributions from the following: AbbVie; Alzheimer's Association; Alzheimer's Drug Discovery Foundation; Araclon Biotech; BioClinica, Inc.; Biogen; Bristol‐Myers Squibb Company; CereSpir, Inc.; Cogstate; Eisai Inc.; Elan Pharmaceuticals, Inc.; Eli Lilly and Company; EuroImmun; F. Hoffmann‐La Roche Ltd and its affiliated company Genentech, Inc.; Fujirebio; GE Healthcare; IXICO Ltd.; Janssen Alzheimer Immunotherapy Research & Development, LLC.; Johnson & Johnson Pharmaceutical Research & Development LLC.; Lumosity; Lundbeck; Merck & Co., Inc.; Meso Scale Diagnostics, LLC.; NeuroRx Research; Neurotrack Technologies; Novartis Pharmaceuticals Corporation; Pfizer Inc.; Piramal Imaging; Servier; Takeda Pharmaceutical Company; and Transition Therapeutics. The Canadian Institutes of Health Research is providing funds to support ADNI clinical sites in Canada. Private sector contributions are facilitated by the Foundation for the National Institutes of Health (www.fnih.org). The grantee organization is the Northern California Institute for Research and Education, and the study is coordinated by the Alzheimer's Therapeutic Research Institute at the University of Southern California. ADNI data are disseminated by the Laboratory for Neuro Imaging at the University of Southern California.
